# The Potential of Isolation Source to Predict Colonization in Avian Hosts: A Case Study in *Campylobacter jejuni* Strains From Three Bird Species

**DOI:** 10.3389/fmicb.2018.00591

**Published:** 2018-03-29

**Authors:** Clara Atterby, Evangelos Mourkas, Guillaume Méric, Ben Pascoe, Helen Wang, Jonas Waldenström, Samuel K. Sheppard, Björn Olsen, Josef D. Järhult, Patrik Ellström

**Affiliations:** ^1^Department of Medical Sciences, Zoonosis Science Center, Uppsala University, Uppsala, Sweden; ^2^Department of Medical Biochemistry and Microbiology, Zoonosis Science Center, Uppsala University, Uppsala, Sweden; ^3^Department of Biology and Biochemistry, The Milner Centre for Evolution, University of Bath, Bath, United Kingdom; ^4^MRC CLIMB Consortium, Bath, United Kingdom; ^5^Centre for Ecology and Evolution in Microbial Model Systems, Linnaeus University, Kalmar, Sweden

**Keywords:** *Campylobacter*, interspecies transmission, colonization, wild bird, mallard, chicken

## Abstract

*Campylobacter jejuni* is the primary cause of bacterial gastroenteritis worldwide, infecting humans mostly through consumption of contaminated poultry. *C. jejuni* is common in the gut of wild birds, and shows distinct strain-specific association to particular bird species. This contrasts with farm animals, in which several genotypes co-exist. It is unclear if the barriers restricting transmission between host species of such specialist strains are related to environmental factors such as contact between host species, bacterial survival in the environment, etc., or rather to strain specific adaptation to the intestinal environment of specific hosts. We compared colonization dynamics *in vivo* between two host-specific *C. jejuni* from a song thrush (ST-1304 complex) and a mallard (ST-995), and a generalist strain from chicken (ST-21 complex) in a wild host, the mallard (*Anas platyrhynchos)*. In 18-days infection experiments, the song thrush strain showed only weak colonization and was cleared from all birds after 10 days, whereas both mallard and chicken strains remained stable. When the chicken strain was given 4 days prior to co-infection of the same birds with a mallard strain, it was rapidly outcompeted by the latter. In contrast, when the mallard strain was given 4 days prior to co-infection with the chicken strain, the mallard strain remained and expansion of the chicken strain was delayed. Our results suggest strain-specific differences in the ability of *C. jejuni* to colonize mallards, likely associated with host origin. This difference might explain observed host association patterns in *C. jejuni* from wild birds.

## Introduction

The majority of human pathogens are zoonotic and able to infect more than one host species (Taylor et al., 2001; Woolhouse et al., 2001), including diseases of significant health concerns such as Salmonellosis, Tuberculosis, Cholera and Lyme disease. Furthermore, host-restricted pathogens are believed to have evolved from ancestors with a generalist life style and in some cases, this has been associated with a change in pathogenicity (Bäumler and Fang, 2013). One example is *Salmonella enterica* serovar Typhi. In contrast to most of the related serovars in the *S. enterica* subspecies that are generalist enteric pathogens, *S*. Typhi separated 10,000 to 70,000 years ago to become a specialist pathogen of humans causing disseminated septicaemia (typhoid fever) (Selander et al., 1990; Roumagnac et al., 2006). The mechanisms behind host specificity for bacterial pathogens are multifactorial and include colonization, replication in the host, and competition with the surrounding microbiota (Zahrt, 1998; Bäumler and Fang, 2013). In some bacterial species, specific genomic alterations are associated with specialist pathogen lineages, reviewed in Bäumler and Fang (2013). Such signatures can involve genomic decay and genomic rearrangements, the result of the accumulation of mutations or rearrangements of genes in the absence of selection pressure to maintain gene function. Lateral gene transfer between bacterial strains or species, can also result in the accumulation of pathogen specific genetic elements that, for example, allow the bacterium to use alternative transmission/infection routes (e.g., acquired binding to new cell types) or adaptation to the host.

An example of a multi-host zoonotic pathogen is *Campylobacter jejuni*, the leading cause of bacterial gastroenteritis in almost all industrialized countries (Food et al., 2014). *C. jejuni* has a broad host range and has been isolated from domestic (Whiley et al., 2013) and wild mammals (Petersen et al., 2001) and several bird species (Kapperud and Rosef, 1983; Waldenström et al., 2002; Colles et al., 2008a,b). It is frequently detected in environmental waters and can even survive in unicellular eukaryotes such as amoebae (Brennhovd et al., 1992; Axelsson-Olsson et al., 2005). The most important transmission route to humans is consumption of contaminated or undercooked food items, especially from poultry (Dingle et al., 2002). Other sources of human *C. jejuni* infections are water, dairy products, and other farm animals, but although the bacterium has several wild animal hosts, the extent of transmission to humans from such sources is less well-studied. Interestingly, chickens are asymptomatically colonized with *C. jejuni*, suggesting commensal adaptations to the chicken gut (Humphrey et al., 2007).

Genetic relatedness and source attribution of *C. jejuni* has been studied using multilocus sequence typing (MLST). This sequence based typing approach allows clustering of genotypes into sequence types (STs) and clonal complexes (CCs) based on the degree of shared alleles at a set of seven house-keeping genes (Dingle et al., 2001). Although ignoring a lot of sequence variation and presence/absence of the accessory genome, MLST has repeatedly shown that certain CCs, such as ST-21 CC and ST-45 CC, are globally distributed in farm animals and are common causes of human infections (Sheppard et al., 2009b; Dearlove et al., 2016). From source attribution studies, we know that genotypes predominating in the food animal niche can also be retrieved from wild animals, especially wild birds (Sheppard et al., 2009a, 2011). On the other hand, there is growing evidence that in wild birds, *C. jejuni* has strong host association and certain genotypes predominate in specific bird species (Broman et al., 2004; Colles et al., 2008a,b; Sheppard et al., 2011; Griekspoor et al., 2013). Hence, in *C. jejuni*, there are both generalist lineages that can colonize a wide range of host animals and specialist lineages restricted to a few host species, and consequently, specialists and generalists seem to co-exist in many host species including both farm animals and wild birds (Colles et al., 2011; Waldenström and Griekspoor, 2014). Compared to *Salmonella* and *Yersinia* spp. the evolutionary relationship between generalist and specialist lineages of *C. jejuni* is less well-understood, as well as the selection pressures behind evolution of specialism or generalism (Sheppard et al., 2014).

Possible explanations to host association of *C. jejuni* genotypes in wild birds could include limited contact between animal species, hence an ecological or behavioral barrier for interspecies transmission. However, there are several examples of wild bird species that share habitat, at least parts of the year, but still do not seem to exchange *C. jejuni* genotypes (Griekspoor et al., 2013). Other possible factors include differences in diet and feeding behavior of different bird species, but data indicate that *C. jejuni* genotypes show less association to the host feeding behavior and more strongly to taxonomy, where related wild bird species tend to more often carry the same, or closely related *C. jejuni* genotypes across large spatial scales (Griekspoor et al., 2013). An alternative explanation would be bacterial adaptation to the intestinal environment of the host, which is likely related to phylogeny. This could include the ability to adhere to and invade intestinal epithelial cells of a particular species, or adaptation to the host immune system and competition with the host's intestinal microbiota. Indeed, there is evidence that specific genera in the host microbiota can reduce colonization resistance to *Campylobacter* (AGISAR WAGoISo, 2011; Bereswill et al., 2011; Haag et al., 2012; Dicksved et al., 2014) suggesting that different microbiota composition between species can constitute barriers for transmission. Such adaptations could have evolved through long periods of co-existence and resulted in *C. jejuni* lineages restricted to taxonomically related birds (Waldenström and Griekspoor, 2014).

If limited contact between wild bird species, or differences in diet or feeding behavior was the reason behind the strong host association, experimental infection of wild birds with *C. jejuni* strains of different origins would probably yield similar colonization patterns. On the other hand, if a *C. jejuni* strain is adapted to the gut of a certain bird species, it would be expected that challenge of a different bird species with that particular strain would result in reduced colonization. Data in support of this was obtained in an infection experiment using the wild European robin (*Erithacus rubecula*) as a host (Waldenström et al., 2010). In this experiment, robins were challenged with two genetically distant *C. jejuni* strains: one strain, isolated from a human patient (ST-48, ST-48 CC) and another strain, isolated from a song thrush (*Turdus philomelos*) (ST-1315, ST-1304 CC). Whereas the song thrush isolate successfully colonized the birds for up to 10 days, the human isolate failed to colonize the birds. However, although taxonomically related to the *Turdus* genera with species frequently carrying *Campylobacter* spp., European robins are infrequent carriers of *C. jejuni* in nature (Waldenström et al., 2002).

To determine if *C. jejuni* isolated from one bird species would incur decreased colonization ability in a different bird host, we used an *in vivo* challenge experiment in a more relevant model species, the mallard (*Anas platyrhynchos*). Mallards have high prevalence of *C. jejuni* in nature, and can carry many different genotypes simultaneously (Colles et al., 2011; Griekspoor et al., 2013; Mohan et al., 2013). *C. jejuni* belonging to many different CCs have been detected in mallards including such commonly found in humans, farm animals, and other wild birds (www.pubmlst.org/campylobacter/, 20151229). We studied colonization in mallards using combinations of single infection and competition experiments with *C. jejuni* strains isolated from three different bird species [song thrush, domestic chicken (*Gallus gallus domesticus*), and mallard]. Genetic relationships between strains were studied by whole genome comparisons both between the three strains and between the pan genomes of the CCs that the strains belonged to. We test the hypothesis that the *C. jejuni* strains differ in colonization ability, with presumed highest ability in those strains with a known genotypic host association with the model host.

## Results

### *C. jejuni* comparative genomics

**Figure 1:**
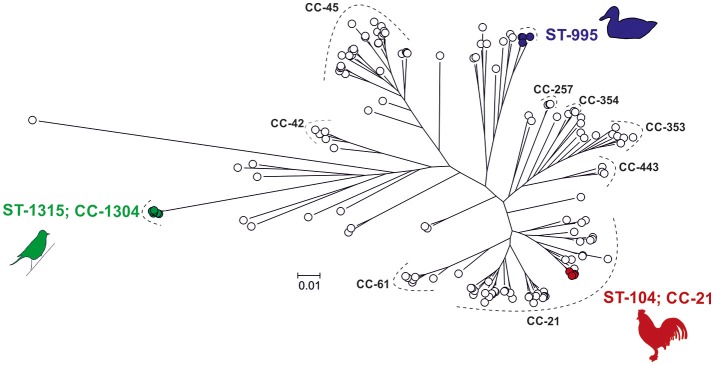
Population structure of 142 *Campylobacter jejuni* strains. Colored *C. jejuni strains* were chosen to represent pan-genomes for the three ST-types used in the study, including ST-995 isolates from mallards (blue), ST-1315 (ST-1304 CC) isolates from song thrushes (green), and ST-104 (ST-21 CC) isolates from broiler chickens (red). One strain from each group was selected for experimental infection of mallards. The phylogenetic tree was reconstructed using an approximation of the maximum-likelihood algorithm in RAXML. The scale bar indicates the estimated number of substitutions per site. Blank circles denote *C. jejuni* genomes added to the analysis to provide phylogenetic context to the strains of interest.

Phylogenetic analysis of 142 strains including the three strains used in the infection experiments (Figure 1, Table S1), revealed that the genetic distance between the song thrush strain and the mallard strain was 1.5 times greater than that between the chicken strain and the mallard strain. By comparing the pan-genomes of the STs of the three strains used for infection using the 142 strains, we identified one ST-specific unique gene out of 1,846 in strain #65 (ST-104, ST-21 complex). Additionally, 14 ST-specific genes out of 4,993 genes were found in the three mallard strains examined and 20 ST-specific genes out of 10,746 genes in the six song thrush strains (Table S2). ST-specific genes were also used as candidates for strain-specific qPCR targets. The specificity of genes *id4678_0651* for the mallard strain, and *id65_1178* for the chicken strain, was confirmed *in vitro* and these targets were subsequently used for the monitoring of strain dynamics during the two competition experimental inoculations of this study.

### Experiment 1: challenge of mallards with the three *C. jejuni* strains in separate groups

**Figure 2:**
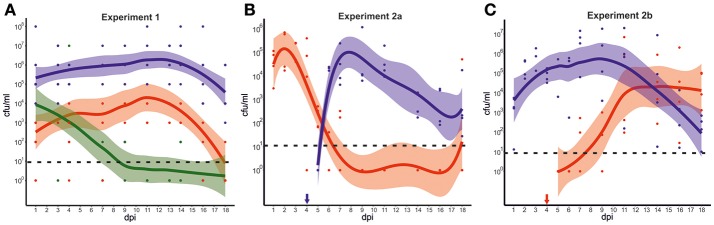
Experimental infection of mallards with *C. jejuni* strains of various hosts. **(A)** Colonization dynamics in mallards during infection with *C. jejuni* strains isolated from mallard (blue), chicken (red), and song thrush (green). The graph illustrates the predicted smoothed mean value for each strain with 95% confidence bands based on the mean colony forming units (cfu) per ml of initial suspension for all fecal samples at each time point, as measured by plate counts. Each dot represents fecal cfu/ml from one bird at each time point. Zeros were replaced for one to fit a log scale. **(B**,**C)** Colonization dynamics in mallards during mixed infection with strains isolated from mallard (blue) and chicken (red). The graphs illustrate the predicted smoothed mean value for each strain with 95% confidence bands corresponding to the mean cfu/ml of initial suspension for all fecal samples at each time point, as determined by real-time PCR with C_T_-values transformed to cfu/ml. In experiment 2a, birds were infected at 0 dpi with the chicken strain followed by the mallard strain at day 4 dpi (indicated by blue arrow). **(B)** In experiment 2b, birds were infected at 0 dpi with the mallard strain followed by the chicken strain at 4 dpi (indicated by red arrow). **(C)** Zeros were replaced for 1 to fit a log scale. The dashed line indicates the theoretical limit of detection.

In experiment 1, each of the three groups of mallards was exposed to one of the three strains from mallard, chicken, or song thrush. As shown in Figure 2A and Table S3, important differences in the dynamics of bacterial colonization were observed between the groups during the experiment. The birds exposed to the mallard strain excreted high numbers of bacteria [mean 10^4^-10^6^ colony forming units per milliliter (cfu/ml)] throughout the experiment, 1–18 days post infection (dpi). The birds exposed to the chicken strain had over all lower levels of bacteria in feces, with peak mean levels of 10^4^ cfu/ml. At 18 dpi, only 2 out of 6 birds exposed to the chicken strain excreted *C. jejuni*. The song thrush strain was detected at 10^3^-10^4^ cfu/ml in feces the first few days after exposure, but bacterial levels declined rapidly. After 7 dpi, the strain could only be detected in two birds and at 18 dpi, the strain was only detected in the caecum of one bird. The mallard strain produced significantly higher bacterial loads, both when analyzing all strains together (Mean_1_, including data from all sampling days, mallard vs. chicken vs. song thrush, *n* = 30; χ = 20.9; df = 2; *p* < 0.0001, Kruskal-Wallis test), and by direct comparison between the mallard strain and the chicken strain or the song thrush strain, respectively (Mean_1_, mallard vs. chicken, *n* = 20, *p* < 0.0002 and mallard vs. song thrush, *n* = 20; *p* = 0.0003, Mann-Whitney test). By direct comparison between the chicken strain and the song thrush strain, the chicken strain produced significantly higher bacterial loads (Mean_1_ chicken vs. song thrush, *n* = 20; *p* = 0.02104, Mann-Whitney test). No *Campylobacter* spp. was detected in fecal samples from the birds prior to inclusion in the experiments. Control experiments were performed to assess the survival of the three *C. jejuni* strains at room temperature in the water used in the experiments. These revealed a rapid loss of viability and none of the strains survived after 12 h. The fractions of each strain surviving in the water after 6 h were 0.40% for the mallard strain, 0.46% for the chicken strain and 0 for the song thrush strain (*SD* = 0.52, 0.56, 0).

### Experiment 2: consecutive challenge with the chicken and mallard strain in the same group

In experiment 2, strain specific colonization ability was further assessed by competition experiments where four birds were challenged consecutively with the chicken and the mallard strains. The experiment was performed twice and in experiment 2a, the chicken strain was introduced at day 0 and the mallard strain at 4 dpi. In experiment 2b the two strains were introduced to a new group of birds in the reverse order. In experiment 2a, the chicken strain had established colonization in all four birds at 1 dpi (Figure 2B). Bacterial numbers started to decrease in 2 birds at 4 dpi, and were markedly reduced in all birds at 5 dpi, 1 day after the introduction of the mallard strain. The chicken strain remained at a level of approximately 10^2^ cfu/ml until 9 dpi, but was thereafter no longer detectable in feces from any of the mallards, except for one bird that again shed high numbers of bacteria at 18 dpi. The mallard strain, introduced at 4 dpi, was detected in high numbers in feces on day 6, and remained high until day 11 dpi. Thereafter it decreased in abundance, but remained at a level of 10^2^-10^3^ cfu/ml until the end of the experiment (Figure 2B).

In experiment 2b, the mallard strain had established colonization of all birds at 1 dpi. In contrast to the chicken strain in experiment 2a, there was no decrease in numbers of the mallard strain at 5 dpi and on. Instead, an increase in bacterial numbers of this strain was observed (Figure 2C, Table S4). The mallard strain remained in high abundance in the fecal samples throughout the experiment. The chicken strain, introduced at 4 dpi, could be detected in low numbers in feces 5 dpi. However, bacterial numbers in fecal samples did not peak until 7 days after inoculation (11 dpi).

In comparisons between experiments, the chicken strain produced significantly higher bacterial loads the first days after introduction in experiment 2a compared to bacterial loads of the same strain the first days after introduction in experiment 2b, when the mallard strain was already present (Chicken 2a Mean_A_, 1–4 dpi vs. Chicken 2b Mean_B_, 5–9 dpi, *n* = 8, *p* = 0.0286, Mann-Whitney test). The corresponding comparison for the mallard strain yielded no significant differences (Mallard 2b Mean_A_, 1–4 dpi, vs. Mallard 2a Mean_B_, *n* = 8; *p* = 0.4857, Mann-Whitney test). Comparisons of fecal bacterial loads between the two strains on the first 4 days when introduced as the second strain, revealed significantly higher bacterial loads of the mallard strain compared to the chicken strain (Mallard 2a Mean_B_, vs. Chicken 2b Mean_B_, *p* = 0.0286, Mann-Whitney test).

### Bacterial colonization at different sites in the mallard gastrointestinal tract

In experiment 1, two birds were sacrificed in each group on 1 and 3 dpi for assessment of bacterial loads at different sites along the gastrointestinal tract. At these time points, all strains were found in high numbers in the gizzard, jejunum, caecum, and colon. On 18 dpi, all remaining birds were sacrificed and bacterial counts were assessed at the same sites of the GI tract. The mallard strain was detected in all GI-segments with high bacterial loads (10^6^-10^8^ cfu in caecum, 10^3^-10^5^ cfu/ml in colon, 10^2^ cfu/ml in gizzard, and 10^3^ cfu/ml in jejunum) (Table 1).

**Table 1:** Detection of the *C. jejuni* strains in different segments of the gastrointestinal tract of infected mallards.

**Experiment 1**										**Experiment 2a**		**Experiment 2b**	
**Strain origin**	**Mallard**	**Chicken**	**Song Thrush**	**Mallard**	**Chicken**	**Mallard**	**Chicken**						
Day (dpi)	1	3	18	1	3	18	1	3	18	18	18	18	18
Gizzard	0/2^a^^,^^b^	1/2	1/6	1/2	0/2	0/6	0/2	0/2	0/6	3/4	0/4	2/4	0/4
Jejunum	1/2	2/2	2/6	0/2	1/2	0/6	1/2	0/2	0/6	2/4	0/4	1/4	0/4
Caecum	2/2	2/2	6/6	2/2	2/2	5/6	2/2	2/2	1/6	4/4	3/4	4/4	4/4
Colon	2/2	2/2	6/6	2/2	2/2	2/6	2/2	2/2	0/6	4/4	1/4	4/4	3/4

a *Number of mallards in which C. jejuni was detected in the indicated segment of the gastrointestinal tract out of the total number of infected mallards investigated at each time point*.

b *The theoretical limit of detection of the assay is 10^1^ cfu/ml*.

In contrast, the chicken strain could not be detected at all in gizzard or jejunum and was found in moderate numbers (10^2^-10^3^ cfu/ml) in 5 out of 6 birds in the caecum and/or colon. Although negative on fecal sampling, 3 birds carried the chicken strain in the caecum at 18 dpi. The song thrush strain was only detected in the caecum of one bird, and this bird was negative on fecal sampling. In experiment 2a and 2b, the mallard strain and chicken strain showed similar bacterial loads in the caecum and colon 18 dpi, but only the mallard strain was detected in jejunum and gizzard (Table 1). Caecum was the preferred site of colonization in both experiment 1 and 2 for all strains (Table 1). In experiment 1, the mallard strain produced significantly higher bacterial loads in caecum compared to all other sites (mallard caecum vs. mallard gizzard, *p* = 0.0001, mallard caecum vs. mallard jejunum, *p* = 0.0001, mallard caecum vs. mallard colon, *p* = 0.0023, Mann-Whitney test). The chicken strain produced significantly higher bacterial loads in caecum compared to gizzard and jejunum (chicken caecum vs. chicken gizzard, *p* = 0.0007, chicken caecum vs. chicken jejunum, *p* = 0.0007, Mann-Whitney test) but there was no significant difference between bacterial loads in cecum and colon (chicken caecum vs. chicken colon, *p* = 0.2310, Mann-Whitney test). For the song thrush strain, there was no significant differences in bacterial loads between the different sites of the GI tract (song thrush caecum vs. song thrush gizzard, *p* = 0.2310, song thrush caecum vs. song thrush jejunum, *p* = 0.0759, song thrush caecum vs. song thrush colon, *p* = 0.5599, Mann-Whitney test).

### General health parameters

All ducks behaved normally and no clinical symptoms of disease were observed in any of the experiments. A slight decrease of body mass was observed in all three experiments during the first 5 days, but after 4 dpi the weight remained stable until the end of the experiments. A similar decrease in body mass was observed also in uninfected mallards when they were moved from the bigger flock into the experimental rooms, and is likely due to handling and settling stress in a new environment. No macroscopic evidence of inflammation or lesions was observed in any of the internal organs during necropsy.

## Discussion

Zoonoses account for the majority of human diseases and many zoonotic pathogens are transmitted from wild to domestic animals and further to humans. Understanding the underlying factors and host–pathogen interactions that determine the outcome of interspecies transmission events for zoonotic, multi-host pathogens is important for adequate planning of interventions to reduce spread to farm animals and ultimately, to protect humans from infection. In this study, we assessed barriers for transmission of *C. jejuni* between bird species. Such knowledge can increase our understanding of the spread of this pathogen from its natural source to domestic animals and further to humans.

Epidemiological evidence suggest very limited interspecies transmission of specialist *C. jejuni* lineages between wild birds, but less is known about the underlying factors behind this observation (Broman et al., 2004; Colles et al., 2008a,b; Sheppard et al., 2011; Griekspoor et al., 2013). We hypothesized that reduced colonization ability due to adaptation to a different host species could account for this pattern and tested this hypothesis by assessing differences in interspecies colonization ability between a generalist and a specialist *C. jejuni* strain isolated from different bird species using captive wild mallards as model host. In single infection experiments, clear differences were observed in colonization ability between the strains, consistent with our expectations from their genetic backgrounds. The mallard strain was the best colonizer with the highest amount of bacteria excreted in the feces throughout the experiment. In contrast, the song thrush isolate showed significantly reduced colonization ability and was only detectable in 2 out of 6 birds after 7 dpi. The chicken strain was secreted throughout the experiment but in significantly lower numbers compared to the mallard strain. These strain specific differences in colonization ability were further emphasized by their relative abundance observed in different parts of the gastrointestinal tract, where the mallard strain could be detected in several parts of the intestine, whereas the two other strains mainly were restricted to caecum and colon.

Although the differences in colonization ability appeared smaller between the chicken- and the mallard strain compared to that between the mallard and the song thrush strain in single infection experiments, the results of the competition experiments revealed a clear competitive advantage of the mallard strain compared to the chicken strain, based on a number of observations. The mallard strain when introduced on day 4 dpi, rapidly outcompeted the chicken strain with peak shedding already 2–3 days after inoculation (Figure 2B), whereas the chicken strain when introduced on day 4 dpi, needed more time to reach peak shedding (Figure 2C). The chicken strain dropped sharply in numbers shortly after the mallard strain was introduced (Figure 2B), but no corresponding decrease of the mallard strain was seen in response to introduction of the chicken strain (Figure 2C). As a whole, fecal shedding of the chicken strain seemed negatively affected by competition with the mallard strain, whereas no significant effect was seen for the mallard strain. Instead, this strain colonized significantly better than the chicken strain under competition, as determined by the relative shedding of the two strains when both were introduced as the second. Some variation in the level of colonization was observed for the same strains between experiment 1 and 2 and these differences were likely due to the fact that colonization was monitored by PCR in experiment 2 as well as differences in the age and number of birds between these experiments. Control experiments assessing the survival of the strains in water suggest that all strains were short lived under the experimental conditions. Although the song thrush strain survived for a shorter time period compared to the other two strains, this did not seem to have any impact on the establishment of initial colonization as mallards inoculated with that strain shed more bacteria 1 dpi compared to those inoculated with the chicken strain. Furthermore, as the water pools and the experiment rooms were cleaned every 24 h, differences in long term bacterial survival in the environment are not likely to have had a large impact on the observed colonization patterns in the birds.

Taken together, these results show clear strain specific differences in the ability to colonize the mallard gastrointestinal tract. The differences correspond well with the phylogenetic relatedness of the strains and are likely associated with host origin. Our genomic analysis revealed a greater genetic distance between the mallard strain and the song thrush strain compared to that between the mallard and the chicken strain. This pattern corroborates what was even more clearly seen in an earlier study using 2,294 *C. jejuni* strains from wild birds, domestic chickens and humans (Griekspoor et al., 2013). Although the number of song thrush and mallard isolates used in the present study was small, comparison of pan-genomes from the CCs of each of the three strains revealed important differences in their gene content. Some of these genes could possibly explain the observed differences in the ability to colonize the mallard gastrointestinal tract. However, an accurate analysis of such gene ontology would require more strains from song thrushes and mallards as well as a rigorous panel of *in vitro* assays. At this point, we can only speculate around possible factors making up the barriers for colonization of the mallard (or any bird's) intestine and which host specific *C. jejuni* genes that are needed to overcome them. The host's immune response is always an important factor in infection and both innate- and humoral immunity is likely involved. However, although the immune response was a likely cause of the reduction in bacterial numbers observed toward the end of the experiments, it is less likely that specific immunity accounted for the dramatic effects seen in the competition experiments or in the rapid reduction of bacterial numbers of the song thrush strain. Instead, our results suggest that other factors related to the intestinal environment of specific bird species might make up such barriers. These could include the intestinal microbiota, structure and glycosylation of the mucin layer, structure of receptors expressed at the epithelial surface or other factors that would favor strains that have co-evolved with its host. Such barriers would lead to lower bacterial load, less shed bacteria and hence, fewer potential transmission events of the new strain. In other words, a reduced fitness of the strain in the population, especially in competition with other strains that are better adapted to the host. Hence, in the absence of competing strains, a less-than-optimally adapted strain may still successfully transmit, but given the competitive landscape of *C. jejuni* in birds, the chance of long-term proliferation is reduced. Consistent with this, it may be expected that a generalist *C. jejuni* genotype would have better colonization ability in a new host compared to a specialist genotype adapted to a different host species, as was the case in this study.

Our study was performed in captive wild mallards, and the results cannot be directly extrapolated to infection of chickens as there are important differences between these species in terms of anatomy, food intake, behavior, etc. Attempts to study differences in colonization ability between *C. jejuni* strains in chickens have been made through infection experiments (Glunder, 1995; Korolik et al., 1998; Stas et al., 1999; Hanel et al., 2004; McCrea et al., 2006; de Haan et al., 2010; Chaloner et al., 2014). However, these studies have mainly focused on *C. jejuni* isolates from farm animals and humans and although results are somewhat conflicting between studies, they collectively suggest that host origin is a less important determinant of colonization ability in chickens when comparing *C. jejuni* isolates from such sources. This can likely be explained by the fact that most farm animals and humans share *C. jejuni* strains with similar or identical genotypes and that intensively reared chickens are susceptible to multiple genotypes of *C. jejuni* (Colles et al., 2008b; de Haan et al., 2010; Griekspoor et al., 2013, 2015). On the other hand, an epidemiological study assessing natural transmission of wild bird associated *C. jejuni* strains to free range broiler chickens, suggested limited or no transmission between these bird species despite the fact that the birds occupied the same ranging area (Colles et al., 2008b). Infection experiments in chickens with *C. jejuni* strains of wild bird origin should be performed to assess the risk of spread of such strains to broilers. This is important as contact with broiler chickens or broiler meat is the most common source of human *Campylobacter* infection. If all *C. jejuni* strains can be readily transmitted to broiler chickens, then the wild bird population constitutes an infinite source of new strains that can feed into the chicken population with increased risk of transmission to humans. Direct transmission of specialist *C. jejuni* lineages from wild birds to humans is rare (Griekspoor et al., 2013), and although the results from the mallard infection experiments in this study cannot be extrapolated to infection in humans, it is possible that reduced ability of such strains to colonize the human gut could be the reason behind this.

In conclusion, we show that experimental infection with *C. jejuni* strains in a natural host result in different colonization outcome depending on the host origin of the strain. These results suggest that the strong host association observed in *C. jejuni* from wild birds is likely not due to the absence of direct or indirect contact between these host species. Instead, the barriers for interspecies transmission may be more directly associated to strain specific differences in colonization ability that are likely related to host origin of the bacterial strain as well as to physiological factors of the host.

### Experimental procedures

#### Bacterial strains and genomes

A total of nine strains were isolated and genome sequenced as part of this study. Three *C. jejuni* strains of different host origin were used in the two bird infection experiments. These included strain #3927 (ST-995) (unassigned CC), isolated from a mallard in Sweden in 2002. This ST-type has previously been isolated from chickens and dogs (www.pubmlst.org/campylobacter/, 20151229). Strain #3926 (ST-1315 in ST-1304 CCs) was isolated from a song thrush captured in Sweden in 2000. This strain is the only reported strain of this ST-type and belongs to a CC that appears restricted to thrushes (Griekspoor et al., 2013). Strain #65 (ST-104, in ST-21 CC), was isolated from a broiler chicken in the UK 2006. Strains of ST-104 have been frequently isolated from poultry, humans, several other animal species and from the environment (www.pubmlst.org/campylobacter/, 20151229). Other strains in this CC have also been found in gulls (www.pubmlst.org/campylobacter/, 20170629). Strains were sampled from song thrushes and mallards between 2000 and 2002. (Table S1). Apart from the strain from chicken isolated prior to this study in the UK, all strains were isolated from wild birds captured at the Ottenby Bird Observatory, Öland, Sweden. A total of 134 *C. jejuni* genomes from two previously published studies (AGISAR WAGoISo, 2011; Sheppard et al., 2013, 2014) (Table S1) were added to the dataset of this study to provide a population-wide phylogenetic context for the strains used for infection experiments, as well as to identify genes that are specific to them but not found in a broader population.

The three bacterial strains used for inoculation were minimally passaged on agar plates during isolation and stored at−80°C until used. Bacterial inocula were prepared from frozen stocks by culture on blood agar plates (Columbia agar II containing 8% [vol/vol] whole horse blood) under microaerobic conditions using GENbox anaer (Biomerieux, Askim, Sweden) with CampyGen 2.5L Atmosphere Generation Systems Packs (Oxoid, Basingstoke, UK) for 24 to 48 h at 42°C. Bacteria were harvested and suspended in PBS (pH 7.4). Optical densities were measured using a UVmini-1240 UV-Vis Spectrophotometer (SHIMADZU, Lidingö, Sweden) and cell densities were adjusted to 1 × 10^9^ colony forming units (cfu)/ml. The bacterial concentrations of the inocula were verified by culture on blood agar plates and were all within the range of 0.6–1.8 × 10^9^ cfu/ml.

#### DNA extraction, genome sequencing, assembly, and archiving

The genome sequences of nine strains were obtained. Briefly, DNA was extracted using the QIAmp DNA Mini Kit (Qiagen, Crawley, UK) according to the manufacturer's instructions. Nucleic acid content was quantified on a Nanodrop spectrophotometer prior to normalization and sequencing. Nextera XT (v3 technology, 250 bp paired-end) libraries were prepared and high-throughput sequencing was performed using an Illumina MiSeq benchtop sequencer (Illumina, San Diego, CA). Short reads were assembled *de novo* using SPAdes (version 3.0.0) and evaluated using QUAST (Bankevich et al., 2012; Guenther et al., 2012). Assembled DNA sequences were uploaded to a web-based database based on the BIGSdb platform (Jolley and Maiden, 2010) which allowed the archiving, whole genome gene-by-gene sequence alignments and prevalence analyses. Novel *C. jejuni* genome sequences are available publicly online (NCBI BioProject: PRJNA415188).

#### Reference pan-genome, phylogenetic tree inference, and qPCR targets

A reference pan-genome list was assembled using a previously published method (Méric et al., 2014) from the whole genomes of 13 isolates. Six of the nine newly sequenced strains were isolated from song thrushes (ST-1304 CC) and the remaining three from mallards (ST-995 CC). These were augmented with four genomes isolated from chicken, cattle and human (ST-21 CC) in order to get a representative number of isolates for each CC used in the pan-genome (Table S1). Briefly, automatic annotations were obtained using RAST (Aziz et al., 2008) and from a total of 22,060 genes detected, 2,489 genes were present in all isolates after the removal of allelic variants using BLAST, with alleles of the same gene being defined as sequenced of >70% sequence identity on >10% of the sequence length (Parkhill et al., 2000; Méric et al., 2014). A whole-genome multiple sequence alignment was obtained by gene-by-gene ortholog identification using MAFFT (Katoh et al., 2002), and concatenation into a single contiguous sequence for input and phylogenetic tree reconstruction using the approximation of the maximum-likelihood algorithm implemented in RAxML (Stamatakis, 2006), running on CLIMB cloud-computing servers (Connor et al., 2016).

Prevalence and allelic variation for every gene of the reference pan-genome list in 142 *C. jejuni* genomes (Table S1) was determined using BLAST, as previously published (Méric et al., 2014, 2015; Pascoe et al., 2015; Yahara et al., 2017). Specifically, genes found in the strains that were used for infection of the birds, and absent in other strains, were considered as candidate targets for development of primers for a quantitative real-time PCR, targeting specifically each of the two strains used in the competition infection experiment. Primers were designed using the online “Primer 3 input software version 0.4.0” (Koressaar and Remm, 2007; Untergasser et al., 2012). After evaluation of several primer candidates two primer pairs were selected (Table 2). The specificity of all primers was assessed by BLAST in the Genbank public repository and evaluated by analysis of fecal samples from *Campylobacter* negative mallards.

**Table 2:** Oligonucleotide primers designed for real-time PCR assay.

**Gene name**	***C. jejuni* strain**	**Designation**	**Sequence**	**PCR product size**	***T*m (°C)**
*id65_1178*	Chicken strain	Fwd-Ch-hypr 241	5′-GTCGTACAGGATTTT ATGATGAGAG-3′	241	61.5
*id65_1178*	Chicken strain	Rev-Ch-hypr 241	5′-CGGCAACTTTTATAA TCAGCTT-3′	241	60.3
*id4678_0651*	Mallard strain	Fwd-Mal-unch 209	5′-CAATCGCCTCTTAAA TCTCCA-3′	209	60.7
*id4678_0651*	Mallard strain	Rev-Mal-unch 209	5′-AAATCTGAATGCGGT GGAAG-3′	209	61.4

#### Mallard infection model and housing

The mallard infection model has been used for studies of influenza A virus, and has been described in detail previously (Järhult et al., 2011). Briefly, 1 day post hatch, male mallards were introduced to the biosecurity level two (BSL2) animal facility at the Swedish National Veterinary Institute (SVA). The mallards were housed indoors with access to pools for swimming and feed and water *ad libitum*. The experimental rooms were HEPA filtered with positive air pressure and double doors and held one pool with water each. Strict hygiene regulations were followed by the staff when handling the mallards and moving between rooms. Before inclusion in the experiments, all birds were tested negative for fecal *Campylobacter* spp. growth on modified charcoal cefoperazone agar (mCCDA) plates (Department of Clinical Microbiology, Uppsala University Hospital).

**Figure 3:**
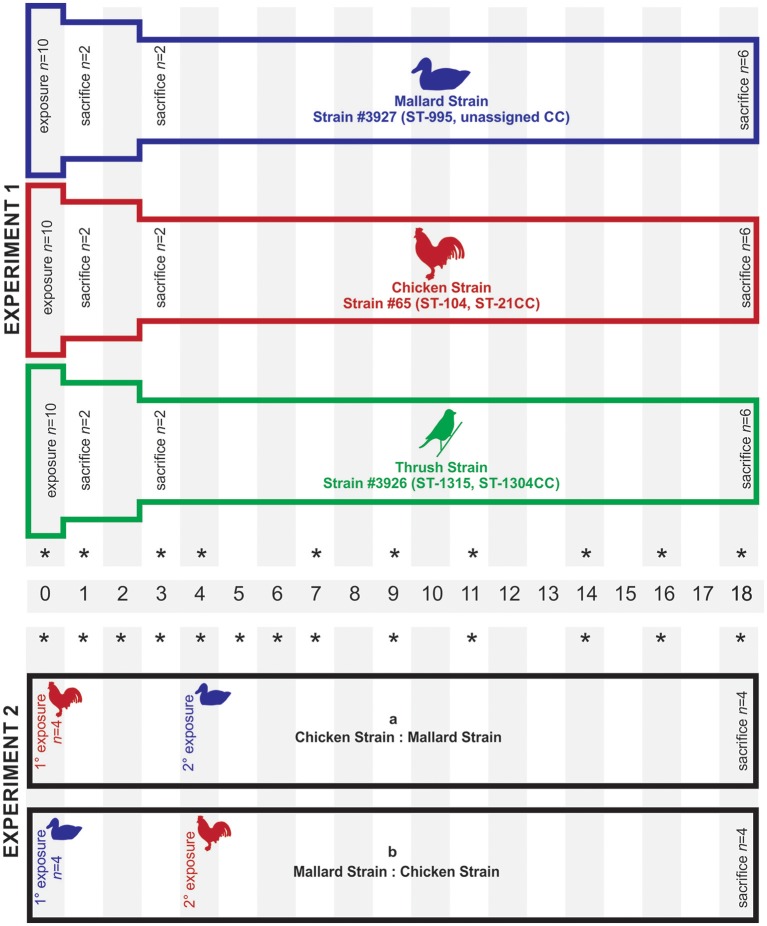
Experimental set-up for mallards (*Anas platyrhynchos)* infected with *C. jejuni* of different host origin. Each bar represents a group of birds infected with *C. jejuni*. In experiment 1, each group of mallards was exposed to *C. jejuni* of different host origin (mallard, chicken, and song thrush) on day 0. In experiment 2, each group was exposed to one *C. jejuni* strain on day 0 and a second *C. jejuni* strain on day 4. In experiment 1, two birds in each group were sacrificed on day 1 and day 3, respectively, while all remaining birds were sacrificed at the end of both experiments (day 18). The stars indicate the sampling days.

Two different experimental setups were applied to study the colonization ability of the *C. jejuni* strains (Figure 3). In experiment 1, 10 mallards (8 weeks of age) were placed in each of three separated experimental rooms. Each group was exposed to one of the three *C. jejuni* strains, mallard (#3927), chicken (#65), and song thrush (#3926) (Table S1), on day 0. Fecal content were obtained from all birds in experiment 1 on 0, 1, 3, 4, 7, 9, 11, 14, 16, and 18 dpi. Experiment 2 was designed to study how the colonization ability of the *C. jejuni* strains of chicken origin (#65) and mallard origin (#3927) was affected by competition with each other within the same bird. Groups of four birds were consecutively infected with the two strains at different time points and the experiment was repeated introducing the strains in the reverse order. In experiment 2a, the birds (24 weeks of age) were exposed to the chicken strain day 0 and to the mallard strain 4 dpi. In experiment 2b, birds (27 weeks of age) were exposed to the mallard strain day 0 and to the chicken strain 4 dpi. Fecal samples were obtained from all birds in experiment 2 on 0, 1, 2, 3, 4, 5, 6, 7, 9, 11, 14, 16, and 18 dpi. In all experiments, exposure was obtained by adding bacterial inoculum to the water pool yielding a bacterial concentration of approximately 5 × 10^4^ cfu/ml of water. The water used was non-chlorinated tap water. During the exposure days, the water pool used for swimming was the only source of drinking water in the experiment room. The pool was emptied and thoroughly rinsed with fresh water every 24 h, including after inoculation. This route of exposure was chosen to simulate a natural situation where birds get infected from contaminated water in their environment. The high bacterial concentration was chosen to make sure that all ducks would ingest viable bacteria. Control experiments were performed to assess the survival of the three strains in water from the same source as used in the animal experiments and at the same temperature (room temperature). Bacterial inocula were prepared as described above and added to 100 ml of water in Erlenmeyer flasks to yield a concentration of appr. 10^4^ cfu/ml. Subsamples of 100 μl were withdrawn at time 0, 6, 12, and 24 h after inoculation and bacterial numbers were assessed by culture on mCCDA plates. The experiment was performed twice with triplicate flasks.

All birds were tagged with color-coded rings for identification. Each bird was visually examined daily for gross signs of injury or lethargy and was subsequently placed in a clean cardboard box where it was weighed and left to defecate. Feces was collected from each box using a sterile cotton swab (ClassiqSwabs, Copan, flock technologies, Täby, Sweden). In experiment 1, two birds from each experimental room were sacrificed on 1 and 3 dpi, and the remaining birds in both experiments were sacrificed on day 18 dpi by euthanasia with pentobarbital injected in the tarsal vein. Necropsies were performed after euthanasia and samples of intestinal contents from the gizzard, jejunum (distal to Meckel's diverticulum), caecum and colon were obtained. All samples were stored on ice in Luria Broth containing 20% glycerol and analyzed for bacterial growth within 4 h.

#### Bacterial quantification in fecal samples

In experiment 1, plate counts were enough to follow the colonization in the three groups as each group was exposed to only one strain of *C. jejuni*. In experiment 2, it was impossible to separate the two strains through phenotypical appearance on the plate and we therefore developed a specific real-time-PCR assay. For bacterial enumeration on agar plates, 100 mg of fecal sample was put into 1 ml of Luria Broth (LB) supplemented with 20% glycerol (to enable freezing of the sample after bacterial enumeration). The sample was vortexed and centrifuged at 400 × g for 1 min to pellet gross fecal material. Samples were diluted in ten-fold dilution series in PBS and 100 μl from each dilution was plated onto mCCDA plates. The plates were incubated microaerobically at 42°C for 48 h before colonies were enumerated. Bacterial concentration in fecal samples was expressed as cfu/ml of the initial suspension (in LB glycerol). Due to the large number of birds in experiment 1, bacterial numbers on plates were estimated to the nearest 10 or 100.

#### Development of real-time-PCR for bacterial quantification

Mallard and chicken strains were identified with real-time PCR using the SsoAdvanced Universal SYBR Green Supermix (Bio Rad Laboratories AB, Sundbyberg, Sweden) on a CFX96 Optics Module C1000 Thermal Cycler (Bio Rad Laboratories AB, Sundbyberg, Sweden). The reaction mixture consisted of 1 x SYBR Green, 0.3 μM of each of the primers (Table 2), 1 μL of template solution and DNase/RNase-free distilled water (Thermo Fischer Scientific, Waltham, MA, U.S.A.) to a final volume of 20 μL. Final cycling conditions were 98°C for 3 min followed by 40 cycles of 98°C for 15 s and 63°C for 60 s, followed by a dissociation curve ranging from 65 to 95°C. All PCR reactions were performed in triplicates. The Bio-Rad CFX Manager 3.1 software (Bio Rad Laboratories AB, Sundbyberg, Sweden) was used for data analyses. The melting point for each amplicon was identified and set as a measure of the specificity of the assay. Primers were designed as described above, and appropriate annealing temperature was assessed using a thermal gradient during optimization. Expected size of the PCR products were verified by agarose gel electrophoresis with a 1.5% Tris-Acetate-EDTA (Sigma-Aldrich AB, Stockholm, Sweden) agarose gel viewed under UV light together with a GeneRuler™ 100 bp Plus DNA Ladder (Thermo Fischer Scientific, Waltham, MA, U.S.A.).

The *C*_T_-values from the real-time PCR were transformed into cfu using standard curves prepared from each strain (Table S5). The mallard and the chicken strain were inoculated into Brucella broth and incubated microaerobically for 24 h at 42°C. The concentrations of the two bacterial stocks were quantified on a Nano Drop 2000c spectrophotometer (Thermo Fisher Scientific, Waltham, MA, U.S.A.) and confirmed by plate counts on blood agar plates. An amount of 1.4 × 10^9^ and 2.6 × 10^9^ cfu was used for DNA extraction from the mallard and chicken strain, respectively. The extracted DNA was serially diluted to generate a standard curve for each strain. The dilution series was included in each 96 well plate that was analyzed in order to allow bacterial quantification as well as to determine the detection limit of the assay. The software constructed slopes of standard curves by linear regression analysis in order to monitor the amplification efficiency and detection sensitivity of every run.

The QIAamp cador Pathogen Mini kit (Qiagen AB, Sollentuna, Sweden) was used for extraction of DNA from the fecal samples. Extraction was performed according to the manufacturer's instructions, with some slight modifications. Briefly, fecal samples were thawed on ice for approximately 1 h, vortexed thoroughly for 1 min to ensure homogeneity, and centrifuged at 500 × g for 1 min to pellet gross fecal material. Three hundred microliters of supernatant was drawn and mixed with 200 mg 0.1 mm silica beads cat. no. 11079101z (BioSpec Products, Bartlesville, OK, U.S.A.) and 800 μl of ASL stool lysis buffer (Qiagen AB, Sollentuna, Sweden). Samples were vortexed briefly, incubated in a heating block at 95°C for 5 min and instantly put on ice for 10 min. This was followed by bead beating in aBio 101 FastPrep FP120-120V disrupter homogenizer (Savant, Illkirch-Graffenstaden, France) for 3 × 20 s at 5,000 rpm, with incubation for 1 min on ice between each cycle. The tubes were then centrifuged at 2,500 × g for 1 min to precipitate beads and solid material and 200 μl of the supernatant was used for further extraction according to the manufacturer's instructions.

There was generally a good correlation between the estimates of bacterial numbers by plate counts and real-time PCR over time (Tables S3, S4). However, the PCR analysis consistently detected one log higher bacterial numbers compared to plate counts. This over estimation was most likely due to the fact that the PCR analysis detected both viable and dead bacteria, in contrast to plate culture. The general level of colonization was lower in experiment 2a compared to 2b as determined both by plate counts and PCR (Figures 2B,C, Tables S3, S4). Although some variation was seen between the ducks, they displayed roughly the same colonization pattern. One duck in experiment 2a did not defecate at 6 and 14 dpi while two ducks in experiment 2b had insufficient amount of feces at 16 and 18 dpi, respectively.

#### Statistical analysis

Graphs were generated with ggplot2 package using the loess smoothing function for R software (Wickham, 2009) and illustrate the predicted smoothed mean value for each strain with 95% confidence bands based on the mean cfu/ml of all fecal samples at each time point, as measured by plate counts (Figure 2). The cfu/ml of each bird at each time point is indicated by dots in the graph. With the exception of a few time points, every bird had a *C. jejuni* cfu/ml count for each sampling day. The mean cfu/ml counts over the course of several days were calculated for each bird to evaluate overall colonization and colonization at the first days after strain exposure (Table 3).

**Table 3:** Sampling days and statistical analysis for experiment 1 and 2^*^.

**Experiment**	**Mean**	**Sampling days**											
Exp 1	Mean_1_	1	^a^	3	4			7	9	11	14	16	18
Exp 1	Mean_2_	1		3	4			7	9	11	14	16	18
Exp 2	Mean_B_	1	2	3	4	5	6	7	9	11	14	16	18
Exp 2	Mean_C_	1	2	3	4	5	6	7	9	11	14	16	18

* *All sampling days are indicated as numbers in the table. Shaded boxes display from which days data were included in each mean value used for statistical analysis. For experiment 1, two mean values were calculated from the fecal cfu count of each of the 30 birds. Mean_1_ was based on data from all sampling days and mean_2_ was based on data from week two and three. For experiment 2, two mean values were calculated from the fecal cfu counts of each of the eight birds. Mean_B_ was based on data from the first four days after introduction of the first C. jejuni strain in experiment 2a and 2b, respectively, and mean_C_ was based on data from the first four days after introduction of the second C. jejuni strain*.

a *No samples were obtained on day 2, 5, and 6 in experiment 1*.

The mean value from each individual bird was grouped with mean values from birds in the same experiment exposed to the same strain. For experiment 1, this resulted in three groups, mallard, chicken and song thrush (*n* = 10/group), and two mean values per group, mean_1_ and mean_2_. For experiment 2, this resulted in two groups, mallard and chicken (*n* = 4/group), and two mean values per group, mean_B_ and mean_C_. The groups were compared using non-parametric Kruskal-Wallis one-way analysis of variance and non-parametric Mann-Whitney test.

For the necropsy cfu counts in experiment 1, every bird had a *C. jejuni* cfu/ml count for each organ (gizzard, jejunum, colon, and caecum). The cfu/ml counts from all birds exposed to the same strain were grouped in the respective organ: gizzard, jejunum, caecum, and colon (*n* = 10/group). The groups were compared using non-parametric Kruskal-Wallis one-way analysis of variance followed by non-parametric Mann-Whitney test. Statistical analysis from experiment 2 was not performed due to the small sample size. Kruskal-Wallis one-way analysis and non-parametric Mann-Whitney tests were performed using GraphPad Prism version 6 and *p*-values < 0.05 were considered significant.

## Ethics statement

All animal experiments were conducted in accordance with regulations provided by the Swedish Board of Agriculture and were approved by the Ethical Committee on Animal Experiments in Uppsala (permit number C20/14). Collection of *Campylobacter* isolates from wild birds at Ottenby Bird Observatory was approved by the Animal Experimentation Committee of Linköping (permit number Dnr112-11).

## Author contributions

CA, JW, JJ, and PE: conceptualization. CA, EM, GM, BP, HW and PE: formal analysis. GM, JW, SS, BO, JJ, and PE: funding acquisition. CA, EM, GM, HW, JJ, and PE: investigation. SS, JJ, and PE: supervision. CA, EM, GM, BP, and PE: writing—original draft preparation. HW, JW, SS, BO, and JJ: writing—review and editing.

### Conflict of interest statement

The authors declare that the research was conducted in the absence of any commercial or financial relationships that could be construed as a potential conflict of interest.
