# Supplementary material for: The Potential of Isolation Source to Predict Colonization in Avian Hosts: A Case Study in Campylobacter jejuni Strains From Three Bird Species
Source: Front Microbiol. 2018 Mar 29;9:591. doi: 10.3389/fmicb.2018.00591 (PMC5884941; doi:10.3389/fmicb.2018.00591)
Supplement: Supplementary file 1 [file Table1.DOCX]

**Table S1. Isolate details.**

| **Isolate** | **ST** | **CC** | **Source** | **Included in reference pan-genome** | **Reference** |
| --- | --- | --- | --- | --- | --- |
| 4 | 45 | 45 | Chicken | - | Sheppard et al. 2013 |
| 13 | 61 | 61 | Cattle | - | Sheppard et al. 2013 |
| 14 | 2381 |  | Environment | - | Sheppard et al. 2013 |
| 22 | 257 | 257 | Chicken | - | Sheppard et al. 2013 |
| 26 | 267 | 283 | Clinical | - | Sheppard et al. 2013 |
| 27 | 604 | 42 | Clinical | - | Sheppard et al. 2013 |
| 28 | 354 | 354 | Clinical | - | Sheppard et al. 2013 |
| 29 | 43 | 21 | Clinical | - | Sheppard et al. 2013 |
| 30 | 1044 | 658 | Clinical | - | Sheppard et al. 2013 |
| 32 | 11 | 45 | Clinical | - | Sheppard et al. 2013 |
| 34 | 262 | 21 | Clinical | - | Sheppard et al. 2013 |
| 36 | 266 | 21 | Clinical | - | Sheppard et al. 2013 |
| 37 | 883 | 21 | Clinical | - | Sheppard et al. 2013 |
| 39 | 2219 | 45 | Chicken | - | Sheppard et al. 2013 |
| 40 | 21 | 21 | Chicken | - | Sheppard et al. 2013 |
| 42 | 42 | 42 | Cattle | - | Sheppard et al. 2013 |
| 43 | 51 | 443 | Chicken | - | Sheppard et al. 2013 |
| 45 | 583 | 45 | Chicken | - | Sheppard et al. 2013 |
| 48 | 206 | 206 | Cattle | - | Sheppard et al. 2013 |
| 49 | 38 | 48 | Cattle | - | Sheppard et al. 2013 |
| 52 | 334 | 45 | Cattle | - | Sheppard et al. 2013 |
| 54 | 267 | 283 | Chicken | - | Sheppard et al. 2013 |
| 55 | 230 | 45 | Clinical | - | Sheppard et al. 2013 |
| 56 | 45 | 45 | Cattle | - | Sheppard et al. 2013 |
| 57 | 334 | 45 | Chicken | - | Sheppard et al. 2013 |
| 59 | 21 | 21 | Cattle | - | Sheppard et al. 2013 |
| 60 | 53 | 21 | Clinical | - | Sheppard et al. 2013 |
| 62 | 21 | 21 | Cattle | - | Sheppard et al. 2013 |
| 63 | 21 | 21 | Chicken | - | Sheppard et al. 2013 |
| 64 | 814 | 661 | Chicken | - | Sheppard et al. 2013 |
| 65 | 104 | 21 | Chicken | Y | Sheppard et al. 2013 |
| 66 | 353 | 353 | Chicken | - | Sheppard et al. 2013 |
| 67 | 354 | 354 | Chicken | - | Sheppard et al. 2013 |
| 68 | 573 | 573 | Chicken | - | Sheppard et al. 2013 |
| 69 | 2568 | 661 | Chicken | - | Sheppard et al. 2013 |
| 70 | 45 | 45 | Chicken | - | Sheppard et al. 2013 |
| 71 | 50 | 21 | Chicken | - | Sheppard et al. 2013 |
| 72 | 50 | 21 | Chicken | - | Sheppard et al. 2013 |
| 73 | 53 | 21 | Chicken | - | Sheppard et al. 2013 |
| 74 | 262 | 21 | Chicken | - | Sheppard et al. 2013 |
| 75 | 266 | 21 | Chicken | - | Sheppard et al. 2013 |
| 77 | 50 | 21 | Chicken | - | Sheppard et al. 2013 |
| 78 | 50 | 21 | Chicken | - | Sheppard et al. 2013 |
| 79 | 11 | 45 | Chicken | - | Sheppard et al. 2013 |
| 80 | 2030 | 257 | Chicken | - | Sheppard et al. 2013 |
| 81 | 1003 | 45 | Chicken | - | Sheppard et al. 2013 |
| 82 | 45 | 45 | Chicken | - | Sheppard et al. 2013 |
| 83 | 354 | 354 | Chicken | - | Sheppard et al. 2013 |
| 84 | 45 | 45 | Chicken | - | Sheppard et al. 2013 |
| 85 | 3583 | 42 | Cattle | - | Sheppard et al. 2013 |
| 86 | 61 | 61 | Cattle | - | Sheppard et al. 2013 |
| 87 | 273 | 206 | Cattle | - | Sheppard et al. 2013 |
| 88 | 270 | 403 | Cattle | - | Sheppard et al. 2013 |
| 89 | 21 | 21 | Cattle | - | Sheppard et al. 2013 |
| 90 | 45 | 45 | Cattle | - | Sheppard et al. 2013 |
| 91 | 45 | 45 | Cattle | - | Sheppard et al. 2013 |
| 92 | 45 | 45 | Cattle | - | Sheppard et al. 2013 |
| 94 | 104 | 21 | Cattle | Y | Sheppard et al. 2013 |
| 96 | 61 | 61 | Cattle | - | Sheppard et al. 2013 |
| 97 | 19 | 21 | Cattle | - | Sheppard et al. 2013 |
| 99 | 206 | 206 | Cattle | - | Sheppard et al. 2013 |
| 100 | 137 | 45 | Cattle | - | Sheppard et al. 2013 |
| 102 | 583 | 45 | Cattle | - | Sheppard et al. 2013 |
| 103 | 334 | 45 | Cattle | - | Sheppard et al. 2013 |
| 104 | 45 | 45 | Cattle | - | Sheppard et al. 2013 |
| 105 | 257 | 257 | Chicken | - | Sheppard et al. 2013 |
| 106 | 51 | 443 | Chicken | - | Sheppard et al. 2013 |
| 107 | 1079 | 573 | Chicken | - | Sheppard et al. 2013 |
| 108 | 574 | 574 | Chicken | - | Sheppard et al. 2013 |
| 109 | 814 | 661 | Chicken | - | Sheppard et al. 2013 |
| 110 | 21 | 21 | Chicken | - | Sheppard et al. 2013 |
| 111 | 45 | 45 | Chicken | - | Sheppard et al. 2013 |
| 112 | 45 | 45 | Chicken | - | Sheppard et al. 2013 |
| 113 | 883 | 21 | Chicken | - | Sheppard et al. 2013 |
| 114 | 230 | 45 | Chicken | - | Sheppard et al. 2013 |
| 116 | 21 | 21 | Clinical | - | Sheppard et al. 2013 |
| 117 | 21 | 21 | Clinical | - | Sheppard et al. 2013 |
| 119 | 45 | 45 | Clinical | - | Sheppard et al. 2013 |
| 122 | 177 | 177 | Starling | - | Sheppard et al. 2013 |
| 124 | 45 | 45 | Starling | - | Sheppard et al. 2013 |
| 125 | 1020 | 682 | Starling | - | Sheppard et al. 2013 |
| 126 | 1033 | 1034 | Goose | - | Sheppard et al. 2013 |
| 127 | 45 | 45 | Goose | - | Sheppard et al. 2013 |
| 128 | 137 | 45 | Goose | - | Sheppard et al. 2013 |
| 129 | 696 | 1332 | Goose | - | Sheppard et al. 2013 |
| 130 | 702 | 702 | Duck | - | Sheppard et al. 2013 |
| 131 | 45 | 45 | Duck | - | Sheppard et al. 2013 |
| 172 | 459 | 42 | Cattle | - | Sheppard et al. 2013 |
| 173 | 4834 | 353 | Chicken | - | Sheppard et al. 2013 |
| 174 | 4835 |  | Chicken | - | Sheppard et al. 2013 |
| 175 | 48 | 48 | Chicken | - | Sheppard et al. 2013 |
| 176 | 791 |  | Chicken | - | Sheppard et al. 2013 |
| 177 | 3504 | 446 | Chicken | - | Sheppard et al. 2013 |
| 178 | 4836 |  | Chicken | - | Sheppard et al. 2013 |
| 179 | 46 | 206 | Clinical | - | Sheppard et al. 2013 |
| 180 | 4837 | 353 | Chicken | - | Sheppard et al. 2013 |
| 181 | 45 | 45 | Chicken | - | Sheppard et al. 2013 |
| 182 | 47 | 21 | Clinical | - | Sheppard et al. 2013 |
| 183 | 4840 | 48 | Chicken | - | Sheppard et al. 2013 |
| 184 | 4883 | 1275 | Clinical | - | Sheppard et al. 2013 |
| 185 | 403 | 403 | Cattle | - | Sheppard et al. 2013 |
| 186 | 52 | 52 | Clinical | - | Sheppard et al. 2013 |
| 187 | 4838 | 353 | Chicken | - | Sheppard et al. 2013 |
| 188 | 443 | 443 | Clinical | - | Sheppard et al. 2013 |
| 189 | 50 | 21 | Clinical | - | Sheppard et al. 2013 |
| 190 | 1962 |  | Clinical | - | Sheppard et al. 2013 |
| 191 | 61 | 61 | Clinical | - | Sheppard et al. 2013 |
| 192 | 572 | 206 | Clinical | - | Sheppard et al. 2013 |
| 193 | 658 | 658 | Clinical | - | Sheppard et al. 2013 |
| 194 | 2274 |  | Clinical | - | Sheppard et al. 2013 |
| 195 | 50 | 21 | Clinical | - | Sheppard et al. 2013 |
| 196 | 475 | 48 | Clinical | - | Sheppard et al. 2013 |
| 197 | 61 | 61 | Clinical | - | Sheppard et al. 2013 |
| 198 | 4839 |  | Clinical | - | Sheppard et al. 2013 |
| 199 | 22 | 22 | Clinical | - | Sheppard et al. 2013 |
| 200 | 5159 | 353 | Clinical | - | Sheppard et al. 2013 |
| 201 | 939 | 353 | Chicken | - | Sheppard et al. 2013 |
| 202 | 982 | 21 | Cattle | - | Sheppard et al. 2013 |
| 203 | 50 | 21 | Chicken | - | Sheppard et al. 2013 |
| 204 | 452 | 353 | Chicken | - | Sheppard et al. 2013 |
| 205 | 5161 | 61 | Cattle | - | Sheppard et al. 2013 |
| 206 | 132 | 508 | Cattle | - | Sheppard et al. 2013 |
| 207 | 122 | 206 | Clinical | - | Sheppard et al. 2013 |
| 208 | 61 | 61 | Cattle | - | Sheppard et al. 2013 |
| 209 | 922 |  | Cattle | - | Sheppard et al. 2013 |
| 210 | 38 | 48 | Cattle | - | Sheppard et al. 2013 |
| 211 | 806 | 21 | Cattle | - | Sheppard et al. 2013 |
| 212 | 677 | 677 | Clinical | - | Sheppard et al. 2013 |
| 213 | 380 |  | Chicken | - | Sheppard et al. 2013 |
| 214 | 220 | 179 | Chicken | - | Sheppard et al. 2013 |
| 257 | 104 | 21 | Clinical | Y | Sheppard et al. 2013 |
| 261 | 104 | 21 | Clinical | Y | Sheppard et al. 2013 |
| 306 | 702 | 702 | Chicken | - | Sheppard et al. 2013 |
| 3926 | 1315 | 1304 | Song Thrush | Y | Waldenstrom et al. 2010 |
| 3927 | 995 |  | Mallard | Y | This study |
| 4571 | 1304 | 1304 | Song Thrush | Y | This study |
| 4588 | 1304 | 1304 | Song Thrush | Y | This study |
| 4590 | 1304 | 1304 | Song Thrush | Y | This study |
| 4636 | 1304 | 1304 | Song Thrush | Y | This study |
| 4654 | 1304 | 1304 | Song Thrush | Y | This study |
| 4678 | 995 |  | Mallard | Y | This study |
| 4679 | 995 |  | Mallard | Y | This study |
