# Supplementary material for: The Potential of Isolation Source to Predict Colonization in Avian Hosts: A Case Study in Campylobacter jejuni Strains From Three Bird Species
Source: Front Microbiol. 2018 Mar 29;9:591. doi: 10.3389/fmicb.2018.00591 (PMC5884941; doi:10.3389/fmicb.2018.00591)
Supplement: Supplementary file 2 [file Table2.DOCX]

**Table S2. List of genes loci belonging to STs from mallards, song thrushes and chickens.**

| Presence | Gene name* | Description |
| --- | --- | --- |
| Mallard | id4581_0415 | hypothetical protein |
| Mallard | id4581_0422 | hypothetical protein |
| Mallard | id4581_0764 | hypothetical protein |
| Mallard | id4581_0933 | Protein ImpG/VasA |
| Mallard | id4581_1037 | hypothetical protein |
| Mallard | id4581_1311 | hypothetical protein |
| Mallard | id4581_1457 | hypothetical protein |
| Mallard | id4678_0654 | Outer membrane protein ImpK/VasF, OmpA/MotB domain |
| Mallard | id4678_0656 | hypothetical protein |
| Mallard | id4678_0664 | hypothetical protein |
| Mallard | id4678_0671 | hypothetical protein |
| Mallard | id4678_0651 | uncharacterized protein ImpA |
| Mallard | id4678_0652 | Type VI secretion lipoprotein/VasD |
| Mallard | id4678_0670 | hypothetical protein |
| Song Thrush | id4571_0076 | CMP-N-acetylneuraminate-beta-galactosamide- alpha-2,3-sialyltransferase (EC 2.4.99.-) |
| Song Thrush | id4571_0079 | Type III restriction-modification system methylation subunit (EC 2.1.1.72) |
| Song Thrush | id4571_0254 | Putative DNA-binding protein |
| Song Thrush | id4571_0255 | Putative DNA-binding protein in cluster with Type I restriction-modification system |
| Song Thrush | id4571_0263 | hypothetical protein |
| Song Thrush | id4571_0264 | Putative efflux protein |
| Song Thrush | id4571_0265 | Putative efflux protein |
| Song Thrush | id4571_0266 | MFS permease (drug) |
| Song Thrush | id4571_0267 | hypothetical protein |
| Song Thrush | id4571_0268 | hypothetical protein |
| Song Thrush | id4571_0269 | hypothetical protein |
| Song Thrush | id4571_0270 | FIG01220323: hypothetical protein |
| Song Thrush | id4571_0271 | hypothetical protein |
| Song Thrush | id4571_0272 | hypothetical protein |
| Song Thrush | id4571_0824 | Type I restriction-modification system, specificity subunit S (EC 3.1.21.3) |
| Song Thrush | id4571_1302 | predicted ATP-dependent endonuclease, OLD family |
| Song Thrush | id4571_1303 | predicted ATP-dependent endonuclease, OLD family |
| Song Thrush | id4571_1371 | FIG00470444: hypothetical protein |
| Song Thrush | id4571_1372 | hypothetical protein |
| Song Thrush | id4588_1117 | predicted ATP-dependent endonuclease, OLD family |
| Song Thrush | id4571_0076 | CMP-N-acetylneuraminate-beta-galactosamide- alpha-2,3-sialyltransferase (EC 2.4.99.-) |
| Song Thrush | id4571_0079 | Type III restriction-modification system methylation subunit (EC 2.1.1.72) |
| Chicken** | id65_1178 | hypothetical protein |

*Gene names, number and order are based on the C. jejuni strain 11168 annotation (1).
**Gene unique to isolate #65 which was used in the infection experiment.

1. Parkhill J, et al. (2000) The genome sequence of the food-borne pathogen Campylobacter jejuni reveals hypervariable sequences. Nature 403(6770):665–668
